# Pregestational Diabetes and Congenital Heart Defects

**DOI:** 10.1055/s-0042-1755458

**Published:** 2022-11-29

**Authors:** Catarina Maduro, Luís Ferreira de Castro, Maria Lúcia Moleiro, Luís Guedes-Martins

**Affiliations:** 1 Instituto de Ciências Biomédicas Abel Salazar, Universidade do Porto, Porto, Portugal; 2 Departamento da Mulher e da Medicina Reprodutiva, Centro Hospitalar do Porto EPE, Centro Materno Infantil do Norte, Largo Prof. Abel Salazar, Porto, Portugal; 3 Unidade de Investigação e Formação, Centro Materno Infantil do Norte, Porto, Portugal; 4 Instituto de Investigação e Inovação em Saúde, Universidade do Porto, Porto, Portugal

**Keywords:** pregestational diabetes, diabetes mellitus, hyperglycemia, congenital heart defects, diabetes pré-gestacional, diabetes melito, hiperglicemia, defeitos cardíacos congênitos

## Abstract

Studies have consistently shown a significant increase in the risk of congenital heart defects in the offspring of diabetic mothers compared with those of nondiabetic pregnancies. Evidence points that all types of pregestational diabetes have the capacity of generating cardiac malformations in a more accentuated manner than in gestational diabetes, and there seems to be an increased risk for all congenital heart defects phenotypes in the presence of maternal diabetes. Currently, the application of some therapies is under study in an attempt to reduce the risks inherent to diabetic pregnancies; however, it has not yet been possible to fully prove their effectiveness. The present review aims to better understand the mechanisms that govern the association between pregestational diabetes and congenital heart defects and how maternal diabetes interferes with fetal cardiac development, as there is still a long way to go in the investigation of this complex process.

## Resumo

Estudos têm demonstrado consistentemente um aumento significativo no risco de defeitos cardíacos congênitos em filhos de mães diabéticas em comparação com os de gestações não diabéticas. Evidências apontam que todos os tipos de diabetes pré-gestacional têm capacidade de gerar malformações cardíacas mais acentuadas do que no diabetes gestacional e parece haver um risco aumentado para todos os fenótipos de cardiopatias congênitas na presença de diabetes materno. Atualmente, está em estudo a aplicação de algumas terapias na tentativa de diminuir os riscos inerentes à gravidez diabética; no entanto, ainda não foi possível comprovar totalmente a sua eficácia. A presente revisão visa compreender melhor os mecanismos que regem a associação entre diabetes pré-gestacional e cardiopatias congênitas e como o diabetes materno interfere no desenvolvimento cardíaco fetal, pois ainda há um longo caminho a percorrer na investigação deste processo complexo.

## Introduction

Congenital heart defects (CHDs), which affect 40,000 births per year in the United States, 1 represent the most prevalent congenital defects. 1 2 3 4 5 6 7 8 In addition, they are a major cause of noninfectious death in infants 7 and convey an increase in healthcare costs, 9 so their prenatal diagnosis through fetal echocardiography is essential. 4 6

It is described that, worldwide, ∼ 130 million women aged between 20 and 49 years old are diagnosed with diabetes mellitus (DM) and ∼ 21 million births are complicated by maternal diabetes (matDM). 7 Statistical data from Europe and the United States report that pregestational diabetes (PGD) affects 0.3% of pregnant women. 5 10 In addition, there has been an increasing rise in its prevalence over time, especially for type II diabetes. 2 4 8

Pregestational diabetes is associated with an increased risk of congenital defects and maternal and perinatal morbidity and mortality. 2 10 It threatens normal fetal cardiac development at several levels, which explains the wide spectrum of associated CHDs, from small structural and/or functional defects to major heart disease, with potential long-term sequelae. 7 9 10 11 12 Some studies point to a three times higher risk of CHD in the offspring of women with PGD compared with the offspring of nondiabetic women. 4 7 8 Similarly, there is a higher prevalence for each CHD phenotype in this population. 1 3

It is known that in pregnancies associated with prior matDM, hyperglycemia acts as a primary teratogen. 2 4 7 9 11 13 Its presence in early stages of the embryonic development of the cardiovascular system promotes the occurrence of embryopathies, culminating in cardiac defects. 1 3 7 10 12 14

Despite the clear role of hyperglycemia, other factors inherent to matDM, namely placental dysfunction, metabolic disorders such as obesity, and increased oxidative stress appear to be players that also modulate the disturbance of cardiogenesis. 4 11 15

Despite the apparent association between PGD as an environmental risk factor for CHDs, 1 3 5 7 8 9 13 there is still a long way to go in the investigation of this complex process and the mechanisms by which matDM interferes with fetal cardiac development. 3 7 9 11 13

## Methods

**Fig. 1:**
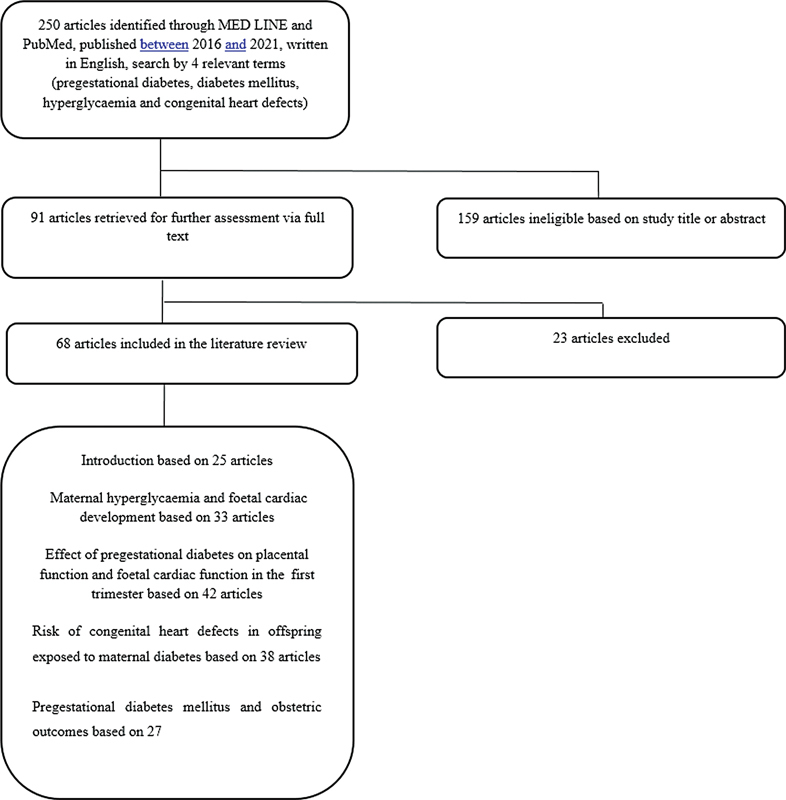
. Flow diagram of the literature review.

The present bibliographic review was based on a literature search of articles published between 2016 and 2021on the PubMed and Medline databases, restricted to articles written in English. Experimental and observational studies involving humans or animals were included. The keywords used were *pregestational diabetes* , *diabetes mellitus* , *hyperglycemia* , and *congenital heart defects* . From the analysis of the abstracts of the articles obtained, those that corresponded to the objective of the review were selected and, additionally, a search of the references of all the analyzed studies was performed to obtain additional information whenever necessary ( Fig. 1 ).

## Maternal Hyperglycemia and Fetal Cardiac Development

The fetal environment in utero influences the development of the fetus during gestation, impacting on the likelihood of developing lifelong disease. Fetal effects resulting from deleterious conditions in utero appear to be proportional to the aggressiveness of these conditions. 16

During pregnancy, changes in glucose metabolism take place, in particular the increase in maternal insulin needs and its resistance in the last trimester, as well as hormones that inhibit its action, stimulating an increase in the amount of insulin supplied by the pancreas. 17 In normal situations, there is the maintenance of a balance of the fetal blood glucose level. At a late stage of gestation, there is a marked drop in glucose uptake in fetal cardiac cells in order to promote proper embryonic cardiogenesis. 18

Several studies highlight a strong correlation between matDM and a significantly increased risk of CHDs in the offspring of affected pregnant woman. 4 7 8 19

Hyperglycemia is the main teratogenic factor in diabetic pregnancies, and its presence prior to conception and in the 1 ^st^ trimester is associated with an increased risk of disturbed embryonic cardiac development. 7 17 20 21 22 23 24 25 Fetal hyperinsulinemia, inherent in pregnancies of diabetic mothers, is also thought to underlie diabetic embryopathy. 26

The spectrum of congenital cardiopathies associated with PGD involves looping, situs anomalies, conotruncal, septal 7 22 and valvular malformations, transposition of great vessels, double-outlet right ventricle, tetralogy of Fallot, 17 24 27 and aortic arch discontinuation. 20 Malformations of the cardiac outflow tract 21 and of the auriculoventricular septum 19 are particularly frequent.

Claudio Gutierrez et al. 23 27 found an association between the hyperglycemic environment during pregnancy and the expansion of the ventricular compartment, decreased area of the ventricular myocardium, and dilation of the ascending aorta in the late stages of pregnancy. Other studies point to a correlation between PGD and hypertrophic fetal cardiomyopathy, 17 24 28 29 possibly due to the hyperinsulinemia that the fetus acquires in the context of maternal hyperglycaemia. 17 28 An evident increase in the thickness of several cardiac structures is also described, especially the interventricular septum, in pregnancies of diabetic mothers compared with fetuses of normal pregnancies, 17 28 resulting in disturbed cardiac function. 17 24 28

Several mechanisms have been implicated in this association between PGD and CHDs. 7 Hyperglycemia plays a role as a promoter of oxidative stress by increasing reactive oxygen and nitrogen species, 7 22 23 27 which will promote the occurrence of genetic changes and abnormalities of the usual pattern of apoptosis in cardiac cells, 23 27 particularly in the neural crest, 17 which is a key part in cardiac development. 7 On the other hand, in an hyperglycemic environment, the alteration of multiple signaling pathways with repercussions in cardiac development is observed: (1) exacerbation of the expression of transforming growth factor beta 1 (TGF-β1), originating an excessive accumulation of extracellular matrix proteins in cardiac tissues; 27 (2) decreased levels of nitric oxide, which is essential for the proper functioning of cardiac endothelial cells and whose reduction leads to inhibition of other signaling pathways dependent on its effect; 22 and (3) excessive stimulation of nucleotide biosynthesis via pentose phosphate, which is responsible for glucose metabolization, preventing proper maturation of cardiac cells. 18 An association between high glucose levels and consequent placental vascular dysfunction due to dysregulation of vascular endothelial growth factor, with a consequent impact on cardiogenesis, has also been described. 17 19 20

It is also known that the association between CHD and PGD does not change according to the type of PGD 3 4 9 or to the type of treatment implemented in the context of diabetes. 3 9 In fact, even with optimal glycemic control, there is an increased risk of developing CHD; 7 17 22 24 27 28 in clinical trials, it has been found that a negligible increase in glucose levels in the mother is associated with defects such as tetralogy of Fallot in the offspring, 7 22 which presupposes that hyperglycemia is potentiated or interacts concomitantly with other conditions in its teratogenic process. 22 Some studies point to a gene-environment interaction in which external factors inherent to the fetal environment may act together with genetic predisposition in modulating cardiac embryogenesis. 7 30

In short, how the fetus reacts to maternal hyperglycemia is subject to several factors, such as the developmental stage in which there was contact with maternal hyperglycemia ( Chart 1 ), its severity, the presence of pathologies or concomitant risk factors, and genetic background, inducing epigenetic changes and a complex interaction with repercussions on fetal cardiogenesis. 7 22

**Chart 1:** Spectrum of congenital cardiopathies associated with pregestational diabetes

Congenital cardiopathies associated with pregestational diabetes
Looping, situs, conotruncal, septal, and valvular anomalies
Transposition of great vessels
Double-outlet right ventricle
Tetralogy of Fallot
Aortic arch discontinuation
Hypertrophic fetal cardiomyopathy
Disturbed cardiac function
Placental vascular dysfunction

## Effect of Pregestational Diabetes on Placental Function and Fetal Cardiac Function in the 1 ^st^ Trimester

Placental development, which takes place in the 1 ^st^ trimester, corresponds to a stage of marked susceptibility, so PGD may be a disruptive factor. 31 32 The interface between the placental vascular system and fetal vessels exposes the placenta to maternofetal endocrine imbalances, with possible harmful repercussions on fetal development. 31 Fetuses exposed to the effects of hyperglycemia have a five-fold increased risk of death in utero. 33 For this reason, diabetic women should be the target of a careful preconceptional assessment and close monitoring from the 1 ^st^ trimester in order to maintain a regular and balanced metabolic control, minimizing the associated risks. 34 35 36 37

Oxygen levels, and consequently reactive oxygen species, are known to increase significantly in the placenta throughout the 1 ^st^ trimester of pregnancy, especially in the presence of PGD, with potential consequences on placental development. It is suggested that this increased oxygen tension amplifies the effects of hyperglycemia at the trophoblast level, culminating in decreased trophoblast proliferation during this period of gestation. As a result, the fetus will receive a deficient nutritional intake, compromising its development. Thus, there seems to be an association between PGD, deficient trophoblast proliferation, and disorders such as fetal growth restriction, pre-eclampsia, and miscarriage. 38

The higher propensity for congenital anomalies in pregnancies of diabetic mothers associated with elevated maternal glucose levels early in gestation is notorious. 39 Maternal hyperglycemia is thought to convey changes in the blood flow established between the mother, the placenta, and the fetus, which may have molecular effects promoting CHD. Placental abnormalities seem to propitiate inflammation and oxidative stress, with disruption of signaling pathways involved in fetal cardiac development. 7 Hyperglycemia is also known to impact proliferation and migration of neural crest cell tissues. 40 These are important for an adequate evolution of fetal cardiac function throughout pregnancy; 40 therefore, this interference in the 1 ^st^ trimester interferes with organogenesis, promoting the appearance of CHDs. 33

Russel et al. demonstrated a higher incidence of fetal cardiac function irregularities in the 1 ^st^ trimester in PGD compared with nondiabetic pregnancies. A deterioration of diastolic function and global cardiac function is noted in this context, highlighting a decrease in the ratio between passive and active ventricular filling and an increase in the isovolumetric relaxation period and in the myocardial performance index. 41 Turan et al. 33 identified a shortening of the isovolumetric contraction period, failure of cardiac contraction capacity, and deterioration of the ejection fraction. It was found that the worse the maternal glycemic control, the greater the deterioration of fetal diastolic function. 33 Sirico et al. 40 also described an increase in the mean 1 ^st^ -trimester fetal heart rate in matDM compared with nondiabetic pregnancies. Some studies seem to indicate that the structural cardiac abnormalities that occur in PGD are noticed after the deterioration of cardiac function shown on ultrasound, raising the suspicion that the latter may occur first. 41

In summary, an adequate functional and structural cardiovascular development of the fetus is determined by the interactions between the maternal, placental, and fetal environments ( Chart 2 ), which are closely dependent on maternal glycemic control in PGD, since glucose levels in the mother influence multiple aspects of fetal cardiogenesis. 33

**Chart 2:** Fetal cardiac dysfunctions in pregestational diabetes and methods for fetal heart function assessment

Fetal cardiac dysfunctions in PGD	Fetal heart function assessment
Deterioration of diastolic function and global cardiac function	Fetal echocardiography
Decrease in the ratio between passive and active ventricular filling	
Increase in the isovolumetric relaxation period and myocardial performance index	
Shortening of the isovolumetric contraction period, failure of cardiac contraction capacity, and deterioration of the ejection fraction	
Increase in the mean 1 ^st^ -trimester fetal heart rate	

Abbreviation: PGD, pregestational diabetes.

## Risk of Congenital Heart Defects in Offspring Exposed to Maternal Diabetes

Clinical trials have demonstrated an increased risk of CHDs in the offspring of diabetic mothers compared with those of nondiabetic mothers. 42 43 44 45 46 47 48 49 What remains to be clarified is the extent of this association, something that differs from study to study, as well as the relationship between matDM and particular subtypes of CHDs, 1 10 42 since the spectrum of associated CHDs seems to encompass > 20 phenotypes. 42

The literature shows that all types of PGD appear to be more likely to cause cardiac malformations than gestational diabetes. 9 42 Similarly, there appears to be an increased risk for all phenotypes of CHDs in the presence of matDM. 1 However, conotruncal defects, auriculoventricular septal defects, heterotaxy, ventricular outflow tract obstruction, and double-outflow right ventricle 10 42 43 have been particularly identified.

It is estimated that the risk of CHD is about three times higher in pregnancies of diabetic mothers compared with those of nondiabetic mothers. 2 8 It is also noteworthy that, among congenital anomalies associated with matDM, CHDs correspond to the most frequent class. 2 9 36 Pregestational diabetes is, therefore, a modifiable risk factor for the incidence of adverse pregnancy outcomes. 9

It is known that the decisive period of fetal cardio genesis is between the 3 ^rd^ and 7 ^th^ weeks of gestation. 1 42 Thus, matDM, by promoting a hyperglycemic environment, generates imbalances in molecular pathways crucial to cardiac embryogenesis, with consequent damage to it. 1 8 36 42 The inherent alterations in insulin resistance favour glucose transfer through the placental interface, promoting a greater secretion of insulin by the pancreas, with increased levels of fetal insulin. 12 Hyperglycemia and subsequent fetal hyperinsulinemia may have teratogenic effects at this early stage of pregnancy. One of its apparent repercussions is myocardial hyperplasia and hypertrophy through insulin receptors on the cardiac surface, which mediate the increase in nutrient synthesis, with subsequent increase in cardiac muscle mass. 10 It has been found that there is an intensification of the expression of these receptors in the presence of poor glycemic control. 12 They are especially numerous in the interventricular septum, which is consistent with the hypertrophy often found in this septum in the offspring of diabetic mothers. 10 Similarly, studies identify an association between interventricular septal thickness and glycated hemoglobin (HgA1c) values. 12 Based on these findings, we conclude that the measurement of HgA1c at preconception and in the 1 ^st^ trimester is crucial for the surveillance of these pregnant women and for the assessment of the risk of congenital malformations. 36

Simultaneously, some authors argue that, following the oxidative stress intrinsic to PGD, there is a decrease in cell proliferation and an increase in apoptosis, as well as suppression of the expression of certain genes, blocking cardiomyocyte maturation and differentiation, inhibiting embryonic cardiac development. Thus, the regeneration potential of cardiac progenitor cells to restore injured cells is affected, which ultimately may also lead to cardiac abnormalities. 50

Although the extent of cardiac involvement is dependent on maternal glycemic control, 10 it has not yet been possible to quantify how current prenatal measures modulate the risk of CHDs. Measures to reduce the risk of cardiac abnormalities in PGD include strict control of blood glucose and body mass index 8 at preconception and in the early stages of pregnancy. In addition, early fetal ultrasound monitoring allows the diagnosis of a part of the cardiac anomalies in the 1 ^st^ half of pregnancy, making it possible to establish a timely course of action in the course of pregnancy. 51

The role of insulin analogues in 1 ^st^ -trimester pregnancies of diabetic mothers is currently under evaluation. In fact, there seems to be a decreased risk of CHDs in the offspring exposed to insulin analogues as opposed to human insulin. 36 The feasibility of stem cell therapies in CHDs is also under discussion, since PGD impairs the biological performance of progenitor cells and cardiac stem cells. 50

In conclusion, the pathogenesis of CHDs remains unclear, but seems to involve multiple players, with a crucial interaction between genetic and environmental factors. 8 42 52 These factors seem to lead to cardiac developmental disorders, both at morphological and functional levels, conditioning a wide spectrum of CHDs. 11 12 Therefore, the study of glycemic control interventions in pregnant women is essential to reduce the risk of these malformations. 36

## Pregestational Diabetes Mellitus and Obstetric Outcomes

Pregnancies complicated by PGD present a greater association with unfavorable maternofetal outcomes compared with pregnancies of nondiabetic mothers, 53 54 55 56 culminating in increased morbidity, 35 57 58 mortality, and hospitalizations. 56

The complications resulting from matDM with greater emphasis in the literature encompass fetal macrosomia, congenital anomalies (previously discussed), and miscarriage. 54 59 60 61 62 Also of note is the increased likelihood that the pregnant woman will suffer from hypertensive disorders, such as pre-eclampsia, or that the fetus will develop complications such as growth restriction, 35 54 jaundice, respiratory disorders, and neonatal hypoglycaemia. 63

It seems that the damage inherent to each of these complications is greater the greater the severity and duration of diabetes, pre-existing comorbidities, and glycemic control in early pregnancy. 35 59 60 Interestingly, even in pregnancies of diabetic mothers with better blood glucose levels, adverse outcomes continue to be recorded, and it remains unclear how much glycemic control effectively mitigates the risks inherent to matDM. On the other hand, maternal hypoglycemia also has the potential to generate adverse effects in pregnancy. Its presence in a fetus usually with high glucose levels seems to be associated with a greater threat of miscarriage. 60

Therefore, the assessment of fetal well-being during pregnancy involves several factors, and amniotic fluid volume is a key tool when we talk about diabetes in pregnancy. In pregnancies of diabetic mothers, there is a correlation between poor glycemic control and excessive accumulation of amniotic fluid (polyhydramnios). 61 The detection of this and other complications involves a multidisciplinary surveillance, with analytical and echographic controls, whose frequency and most effective management is still to be clarified, since all of them have limitations. 55 60 64 65

Since the obstetric prognosis is largely influenced by the follow-up implemented in diabetic mothers, 54 it would be ideal to initiate a line of preconception care. This would include closer monitoring of diabetic women who are planning to become pregnant in the near future, making efforts to control blood glucose values prior to pregnancy and implementing a multidisciplinary approach to optimize care, 58 66 which should include the regular screening for nephropathy and retinopathy and the verification of potentially teratogenic prescribed drugs, among other measures, in order to reduce as much as possible the risk of complications during pregnancy. 58

Despite advances in glycemic control and prenatal surveillance, improving obstetric care in this population remains a challenge: not all patients have access to healthcare and a large proportion do not use preconception care, missing a key window of opportunity to institute effective disease control before pregnancy to prevent or mitigate adverse outcomes ( Chart 3 ). 66

**Chart 3:** Some methods for the assessment of fetal well-being in pregestational diabetes

Methods for the assessment of fetal well-being in PDG
Analytical controls (glycaemia, serum levels of Pregnancy-Associated Plasma Protein A [PAPP-A]…)
Fetal ultrasound monitoring
Amniotic fluid volume
Placental vascularization indices (uterine artery pulsatility levels…)

Abbreviation: PGD, pregestational diabetes.

## Discussion

The incidence of CHDs is clearly higher in the offspring of mothers with PGD compared with in the offspring of nondiabetic women, 1 3 4 7 8 with malformations of the cardiac outflow tract 21 and of the auriculoventricular septum 19 being particularly frequent. There is an association between PGD and fetal hypertrophic cardiomyopathy, 17 24 28 29 with an evident increase in the thickness of cardiac structures such as the interventricular septum, 17 28 leading to negative effects on long-term cardiac function. 17 24 28

Hyperglycemia is identified as the primary teratogen in this relationship, 2 4 7 9 11 13 and its presence in the early stages of cardiac embryogenesis seems to favour the occurrence of CHDs. 1 3 7 10 12 14 In addition, other factors inherent to matDM, such as placental dysfunction, increased oxidative stress, and alteration of multiple molecular signaling pathways appear to be players that also negatively modulate cardiogenesis. 4 11 15

**Fig. 2:**
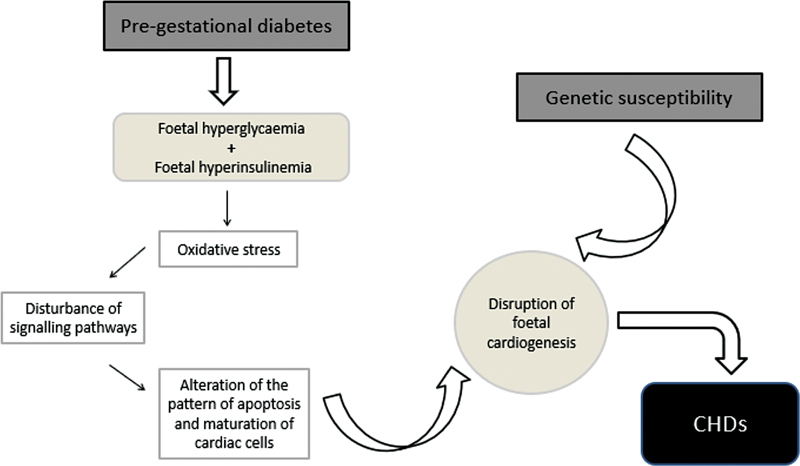
Evidence points to an association between pregestational diabetes and a higher propensity of the offspring to develop congenital heart disease (CHD). This correlation seems to be justified, on the one hand, by the presence of fetal hyperglycemia and hyperinsulinemia and, on the other hand, by a deficient placental development. Thus, the teratogenesis of maternal diabetes will reside in the generation of reactive oxygen and nitrogen species (oxidative stress), culminating in epigenetic and cell cycle changes, which condition a defective cardiogenesis. Simultaneously, studies highlight the role of genetic predisposition for abnormal fetal cardiac development, so that this interrelation between fetal environment and genetic background will be at the basis of fetal heart defects.

Thus, cardiac abnormalities in the context of matDM have a multifactorial basis, highlighting the gene-environment interaction; that is, environmental factors, such as PGD, act together with genetic predisposition in modulating cardiovascular development 7 30 ( Fig. 2 ).

How the fetus reacts to maternal hyperglycemia depends on several factors, such as the developmental stage in which it came into contact, its severity, the presence of concomitant diseases, and genetic background. This interaction results in epigenetic changes with considerable repercussions on fetal cardiogenesis. 7 22 Research in this area shows an important correlation between CHDs and maternal blood glucose levels at an early stage of pregnancy; therefore, the risk of CHD increases in pregnancies based on poor glycemic control or with repeated episodes of acute complications of diabetes at an earlystage. 4 8 9

Furthermore, pregnancies complicated by PGD are more associated with unfavorable maternal and fetal outcomes 53 54 55 56 and higher fetal and maternal morbidity and mortality. 35 57 58 At the fetal level, complications involve macrosomia, congenital anomalies, miscarriage, 54 59 60 61 62 shoulder dystocia or contusions at delivery, 53 64 jaundice, respiratory disorders, and neonatal hypoglycaemia. 63 For the mother, there is a higher risk of hypertensive disorders 35 54 and higher rates of caesarean sections or perineal injuries. 53 64

There are also several characteristics that, when present in pregnancies of diabetic mothers, are imminently promoters of perinatal mortality, namely a low socioeconomic status, smoking, advanced maternal age, obesity, or twin pregnancies. 31 61 67 68

Early fetal ultrasound monitoring with a set of diagnostic and prognostic markers, such as amniotic fluid volume assessment and fetal echocardiography, allows the identification of some complications and some cardiac anomalies. This surveillance is essential to define an appropriate course of action and to plan the eventual intervention required after birth. 51

The uterine environment experienced by the fetus clearly influences its development during pregnancy and, possibly, will also have repercussions in adulthood. Thus, diabetic women should receive individualized care, ideally from preconception, in order to maintain regular metabolic control and minimize the associated risks. 34 35 36 37

It is necessary to implement a continuous improvement of preconceptional and prenatal care, since there are still women who do not benefit from it, losing the possibility to prevent or mitigate deleterious outcomes. 66 In addition, it is necessary to continue to implement and improve surveillance and intervention programs to address the complications that arise in the context of maternal mortality, since the prevalence of PGD is increasing. 9

Although various resources exist for the early diagnosis of some of the complications of pregnancy in diabetic women, constant research into new markers is crucial, as the current methods have limitations. 55 60 64 65

There is still a significant list of answers to be found: why the teratogenesis associated with hyperglycemia has a more profound impact on certain organs; why the risk of CHDs in pregnancies of diabetic women does not equal the same risk in nondiabetic women, despite optimal glycemic control; or what mechanisms explain the existence of pregnancies in the context of matDM, which record much higher HgA1c values than what is considered acceptable for a pregnancy without birth defects and that, despite this, follow a normal course. 4

In fact, the extent of cardiac impairment is found to be partly dependent on maternal glycemic control, 10 but it is not yet possible to quantify how current prenatal measures modulate the risk of CHDs in this setting. 8

In this scenario, the question that arises is what should be the HgA1c threshold considered adequate for a woman with PGD to become pregnant without increasing risks, which remains unanswered. 4

Further studies will be needed to understand how this gene-environment interface occurs and why infants who have been exposed to teratogenic agents such as hyperglycemia are vulnerable to fetal cardiac development disorders. 7 Genetic mechanisms that potentiate susceptibility to certain environmental factors may be involved, something that will need to be clarified in future investigations. 7

Some treatments for diabetic pregnant women are currently under investigation, such as insulin analogues, which, compared to the use of human insulin, appear to have a superior ability to maintain more adequate blood glucose levels. Future investigations should test whether they effectively minimize the risk of CHDs in offspring exposed to them. 36 New therapies under study include the use of stem cells, given the role of maternal diabetes in cardiomyocyte development and repair; however, their efficacy has not yet been proven. 50

## Conclusion

Pregestational diabetes has an irrefutable negative influence on pregnancy and fetal cardiac development, even in women with adequate glycemic control. Given the increase of women with this condition in recent years, a proactive attitude is imperative in the information, prevention, and metabolic control of these patients in order to minimize the associated disorders and complications. It is necessary to continue research in this area in order to understand the various aspects of the association between maternal diabetes and fetal cardiac anomalies so that we can have an early and effective intervention in its development and prenatal detection.
